# Supplementary material for: Digital intervention mylovia improves sexual functioning in women with sexual dysfunction in randomized controlled trial
Source: NPJ Digit Med. 2026 Feb 3;9:115. doi: 10.1038/s41746-026-02385-z (PMC12868880; doi:10.1038/s41746-026-02385-z)
Supplement: Supplementary file 1 — Revised_Supplementary [file 41746_2026_2385_MOESM1_ESM.pdf]

Supplementary Table 1. Relevant demographic and treatment characteristics over the course of the clinical investigation.

	Control	*mylovia*	Statistical comparison
**T1**	n = 119	n = 114	
**Partnership status (n [%])**			$\chi^2 = 0.25$, $p = 0.615$
single	17 (14.3)	19 (16.7)	
in a relationship	102 (85.7)	95 (83.3)	
**Currently pregnant (n [%])**	3 (2.5)	1 (0.9)	$\chi^2 = 0.21$, $p = 0.645$
**Currently in psychotherapy (n [%])**	29 (24.4)	24 (21.1)	$\chi^2 = 0.36$, $p = 0.546$
Currently in psychotherapy due to sexual dysfunction/sexual pain disorder (n [%])	4 (3.4)	3 (2.6)	$\chi^2 = 0$, $p = 1$
Currently in psychotherapy due to another issue (n [%])	25 (21.0)	21 (18.4)	$\chi^2 = 0.25$, $p = 0.620$
**Currently taking any psychotropic medication[a] (n [%])**	14 (11.8)	13 (11.4)	$\chi^2 = 0.01$, $p = 0.931$
**Regular medication (multiple answers possible; n [%])**			
Antipsychotics	2 (1.7)	0 (0)	$\chi^2 = 0.46$, $p = 0.497$
Anxiolytics	0 (0)	0 (0)	n/a
Hypnotics and sedatives	0 (0)	0 (0)	n/a
Antidepressants	12 (10.1)	12 (10.5)	$\chi^2 = 0.01$, $p = 0.912$
Psychostimulants	2 (1.7)	1 (0.9)	$\chi^2 = 0$, $p = 1$
**Medication as needed (multiple answers possible; n [%])**			
Antipsychotics	0 (0)	1 (0.9)	$\chi^2 = 0.00$, $p = 0.983$
Anxiolytics	0 (0)	0 (0)	n/a
Hypnotics and sedatives	0 (0)	0 (0)	n/a
Antidepressants	1 (0.8)	2 (1.8)	$\chi^2 = 0$, $p = 0.970$
Psychostimulants	0 (0)	0 (0)	n/a

	Control	*mylovia*	Statistical comparison
**T2**	n = 113	n = 106	
**Partnership status (n [%])**			$\chi^2 = 0.33$, $p = 0.566$
single	18 (15.9)	20 (18.9)	
in a relationship	95 (84.1)	86 (81.1)	
**Currently pregnant (n [%])**	5 (4.4)	1 (0.9)	$\chi^2 = 1.35$, $p = 0.245$
**Currently in psychotherapy (n [%])**	30 (26.5)	22 (20.8)	$\chi^2 = 1.01$, $p = 0.314$
Currently in psychotherapy due to sexual dysfunction/sexual pain disorder (n [%])	3 (2.7)	2 (1.9)	$\chi^2 = 0$, $p = 1$
Currently in psychotherapy due to another issue (n [%])	27 (23.9)	20 (18.9)	$\chi^2 = 0.82$, $p = 0.365$
**Currently taking any psychotropic medication[a] (n [%])**	13 (11.5)	13 (12.3)	$\chi^2 = 0.03$, $p = 0.862$
**Regular medication (multiple answers possible; n [%])**			
Antipsychotics	2 (1.8)	0 (0.0)	$\chi^2 = 0.44$, $p = 0.506$
Anxiolytics	0 (0)	0 (0)	n/a
Hypnotics and sedatives	0 (0)	0 (0)	n/a
Antidepressants	11 (9.7)	11 (10.4)	$\chi^2 = 0.03$, $p = 0.874$
Psychostimulants	3 (2.7)	1 (0.9)	$\chi^2 = 0.19$, $p = 0.660$
**Medication as needed (multiple answers possible; n [%])**			
Antipsychotics	0 (0)	0 (0)	n/a
Anxiolytics	0 (0)	0 (0)	n/a
Hypnotics and sedatives	1 (0.9)	2 (1.9)	$\chi^2 = 0$, $p = 0.956$
Antidepressants	0 (0)	0 (0)	n/a
Psychostimulants	0 (0)	0 (0)	n/a

[a] ATC classification codes N05 / N06.

Self-compiled questionnaire on cognitions and behaviors related to sexual pain (translated into English)

Instructions: Women have various thoughts and feelings about vaginal penetration. Vaginal penetration means the insertion of a penis, finger (yours or your partner's), tampon, and/or another object into the vagina. This questionnaire asks about vaginal penetration in the last four weeks and about your thoughts regarding vaginal penetration.

You will be asked to indicate to what extent each of these statements applies to you. Your thoughts about vaginal penetration are important, and you can complete this questionnaire fully, regardless of whether penetration is possible/has ever been possible for you or not. One of the questions also concerns the topic of partnership. Please answer this question according to how you generally think - regardless of whether you are currently in a relationship or not.

There are no “good” or “bad” answers to the questions or statements. Your answer is the best when it reflects your own thoughts and feelings regarding vaginal penetration.

1) To what extent was vaginal penetration during sexual intercourse (e.g., insertion of a penis or dildo into your vagina) possible in the past four weeks?
2) To what extent was the vaginal insertion of one or more finger(s) possible in the past four weeks?
3) To what extent was the vaginal insertion of a tampon or dilator possible in the past four weeks?

Answer options: 0 = not attempted, 1 = attempted, but not successful, 2 = attempted and sometimes successful, 3 = attempted and always successful

4) Have you experienced pain during (the attempt of) vaginal penetration in the past four weeks?

Answer options: 0 = never, 1 = sometimes, 2 = regularly, 3 = mostly, 4 = always

5) How severe was the pain during (the attempt of) vaginal penetration?

Scale from 0-10; anchors: 0 = no pain, 10 = worst imaginable pain

6) It feels frightening not knowing what happens in my body during penetration.
7) I fear I can't do anything to change the pain during penetration.
8) I am a poor partner when penetration fails.
9) Penetration will feel good.
10) I am afraid that my vagina is too narrow for penetration.

Scale from 0-10; anchors: 0 = does not apply at all, 6 = applies very strongly

Scoring:

Questions 1-3:	Question 4:	Question 5:	Questions 6-8 and 10:	Question 9:
0 = 100	0 = 0	0 = 0	0 = 0	0 = 100
1 = 100	1 = 25	1 = 10	1 = 16.66	1 = 83.33
2 = 50	2 = 50	2 = 20	2 = 33.33	2 = 66.66
3 = 0	3 = 75	3 = 30	3 = 50	3 = 50
	4 = 100	4 = 40	4 = 66.66	4 = 33.33
		5 = 50	5 = 83.33	5 = 16.66
		6 = 60	6 = 100	6 = 0
		7 = 70		
		8 = 80		
		9 = 90		
		10 = 100		

Total Score: Mean of items 1-10

Total Scale Range: 0-100

Section/topic	No	CONSORT 2025 checklist item description	Reported on page no.
**Title and abstract**			
Title and structured abstract	1a	Identification as a randomised trial	1
	1b	Structured summary of the trial design, methods, results, and conclusions	2
**Open science**			
Trial registration	2	Name of trial registry, identifying number (with URL) and date of registration	21
Protocol and statistical analysis plan	3	Where the trial protocol and statistical analysis plan can be accessed	n/a can be provided if needed
Data sharing	4	Where and how the individual de-identified participant data (including data dictionary), statistical code and any other materials can be accessed	n/a can be provided upon reasonable request
Funding and conflicts of interest	5a	Sources of funding and other support (eg, supply of drugs), and role of funders in the design, conduct, analysis and reporting of the trial	36
	5b	Financial and other conflicts of interest of the manuscript authors	36-37
**Introduction**			
Background and rationale	6	Scientific background and rationale	3-5
Objectives	7	Specific objectives related to benefits and harms	5-6
**Methods**			
Patient and public involvement	8	Details of patient or public involvement in the design, conduct and reporting of the trial	n/a
Trial design	9	Description of trial design including type of trial (eg, parallel group, crossover), allocation ratio, and framework (eg, superiority, equivalence, non-inferiority, exploratory)	21
Changes to trial protocol	10	Important changes to the trial after it commenced including any outcomes or analyses that were not prespecified, with reason	n/a
Trial setting	11	Settings (eg, community, hospital) and locations (eg, countries, sites) where the trial was conducted	21
Eligibility criteria	12a	Eligibility criteria for participants	22
	12b	If applicable, eligibility criteria for sites and for individuals delivering the interventions (eg, surgeons, physiotherapists)	n/a
Intervention and comparator	13	Intervention and comparator with sufficient details to allow replication. If relevant, where additional materials describing the intervention and comparator (eg, intervention manual) can be accessed	23-25
Outcomes	14	Prespecified primary and secondary outcomes, including the specific measurement variable (eg, systolic blood pressure), analysis metric (eg, change from baseline, final value, time to event), method of aggregation (eg, median, proportion), and time point for each outcome	25-26
Harms	15	How harms were defined and assessed (eg, systematically, non-systematically)	27
Sample size	16a	How sample size was determined, including all assumptions supporting the sample size calculation	27
	16b	Explanation of any interim analyses and stopping guidelines	n/a
Randomisation:			23
Sequence generation	17a	Who generated the random allocation sequence and the method used	

	17b	Type of randomisation and details of any restriction (eg, stratification, blocking and block size)	23
			**Reported on page no.**
Allocation concealment mechanism	18	Mechanism used to implement the random allocation sequence (eg, central computer/telephone; sequentially numbered, opaque, sealed containers), describing any steps to conceal the sequence until interventions were assigned	23
Implementation	19	Whether the personnel who enrolled and those who assigned participants to the interventions had access to the random allocation sequence	23
Blinding	20a	Who was blinded after assignment to interventions (eg, participants, care providers, outcome assessors, data analysts)	23
	20b	If blinded, how blinding was achieved and description of the similarity of interventions	n/a
Statistical methods	21a	Statistical methods used to compare groups for primary and secondary outcomes, including harms	27-28
	21b	Definition of who is included in each analysis (eg, all randomised participants), and in which group	27-28
	21c	How missing data were handled in the analysis	27-28
	21d	Methods for any additional analyses (eg, subgroup and sensitivity analyses), distinguishing prespecified from post hoc	16-17, 28
**Results**			
Participant flow, including flow diagram	22a	For each group, the numbers of participants who were randomly assigned, received intended intervention, and were analysed for the primary outcome	6-7
	22b	For each group, losses and exclusions after randomisation, together with reasons	6-7
Recruitment	23a	Dates defining the periods of recruitment and follow-up for outcomes of benefits and harms	21-22
	23b	If relevant, why the trial ended or was stopped	n/a
Intervention and comparator delivery	24a	Intervention and comparator as they were actually administered (eg, where appropriate, who delivered the intervention/comparator, how participants adhered, whether they were delivered as intended (fidelity))	n/a
	24b	Concomitant care received during the trial for each group	n/a
Baseline data	25	A table showing baseline demographic and clinical characteristics for each group	9, Supplement
Numbers analysed, outcomes and estimation	26	For each primary and secondary outcome, by group:<br>● the number of participants included in the analysis<br>● the number of participants with available data at the outcome time point<br>● result for each group, and the estimated effect size and its precision (such as 95% confidence interval)<br>● for binary outcomes, presentation of both absolute and relative effect size	13
Harms	27	All harms or unintended events in each group	14-15
Ancillary analyses	28	Any other analyses performed, including subgroup and sensitivity analyses, distinguishing pre-specified from post hoc	10-12, 15-17
**Discussion**			
Interpretation	29	Interpretation consistent with results, balancing benefits and harms, and considering other relevant evidence	17-20
Limitations	30	Trial limitations, addressing sources of potential bias, imprecision, generalisability, and, if relevant, multiplicity of analyses	17, 20

Citation: Hopewell S, Chan AW, Collins GS, Hróbjartsson A, Moher D, Schulz KF, et al. CONSORT 2025 Statement: updated guideline for reporting randomised trials. BMJ. 2025; 388:e081123. https://dx.doi.org/10.1136/bmj-2024-081123
© 2025 Hopewell et al. This is an Open Access article distributed under the terms of the Creative Commons Attribution License (https://creativecommons.org/licenses/by/4.0/), which permits unrestricted use, distribution, and reproduction in any medium, provided the original work is properly cited.

*We strongly recommend reading this statement in conjunction with the CONSORT 2025 Explanation and Elaboration and/or the CONSORT 2025 Expanded Checklist for important clarifications on all the items. We also recommend reading relevant CONSORT extensions. See www.consort-spirit.org.
